# Bridging the gap: Insights in the immunopathology of Lyme borreliosis

**DOI:** 10.1002/eji.202451063

**Published:** 2024-10-13

**Authors:** Marijn E. Snik, Noor E.I.M. Stouthamer, Joppe W. Hovius, Melissa M.J. van Gool

**Affiliations:** ^1^ Center for Experimental and Molecular Medicine, Amsterdam UMC University of Amsterdam Amsterdam the Netherlands; ^2^ Amsterdam Institute for Immunology and Infectious Diseases Amsterdam the Netherlands; ^3^ Division of Infectious Diseases Department of Internal Medicine Amsterdam UMC Multidisciplinary Lyme borreliosis Center Amsterdam UMC University of Amsterdam Amsterdam the Netherlands

**Keywords:** Autoimmunity, *Borrelia burgdorferi*, Immune dysregulation, Inflammation, Lyme borreliosis, Th1/Th2 Balance

## Abstract

Lyme borreliosis (LB), caused by *Borrelia burgdorferi* sensu lato (Bbsl) genospecies transmitted by *Ixodes* spp. ticks, is a significant public health concern in the Northern Hemisphere. This review highlights the complex interplay between Bbsl infection and host–immune responses, impacting clinical manifestations and long‐term immunity. Early localized disease is characterized by erythema migrans (EM), driven by T‐helper 1 (Th1) responses and proinflammatory cytokines. Dissemination to the heart and CNS can lead to Lyme carditis and neuroborreliosis respectively, orchestrated by immune cell infiltration and chemokine dysregulation. More chronic manifestations, including acrodermatitis chronica atrophicans and Lyme arthritis, involve prolonged inflammation as well as the development of autoimmunity. In addition, dysregulated immune responses impair long‐term immunity, with compromised B‐cell memory and antibody responses. Experimental models and clinical studies underscore the role of Th1/Th2 balance, B‐cell dysfunction, and autoimmunity in LB pathogenesis. Moreover, LB‐associated autoimmunity parallels mechanisms observed in other infectious and autoimmune diseases. Understanding immune dysregulation in LB provides insights into disease heterogeneity and could provide new strategies for diagnosis and treatment.

## Graphical abstract

Lyme borreliosis is caused by *Borrelia burgdorferi* sensu lato transmitted by *Ixodes* ticks. The disease progresses from early localized infection to more severe conditions driven by ongoing inflammation. Understanding the role of Th1/Th2 balance, B‐cell dysfunction, and autoimmunity in Lyme borreliosis pathogenesis could improve diagnosis and treatment strategies.

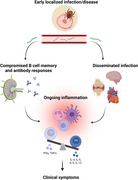

## Introduction

**Figure 1:**
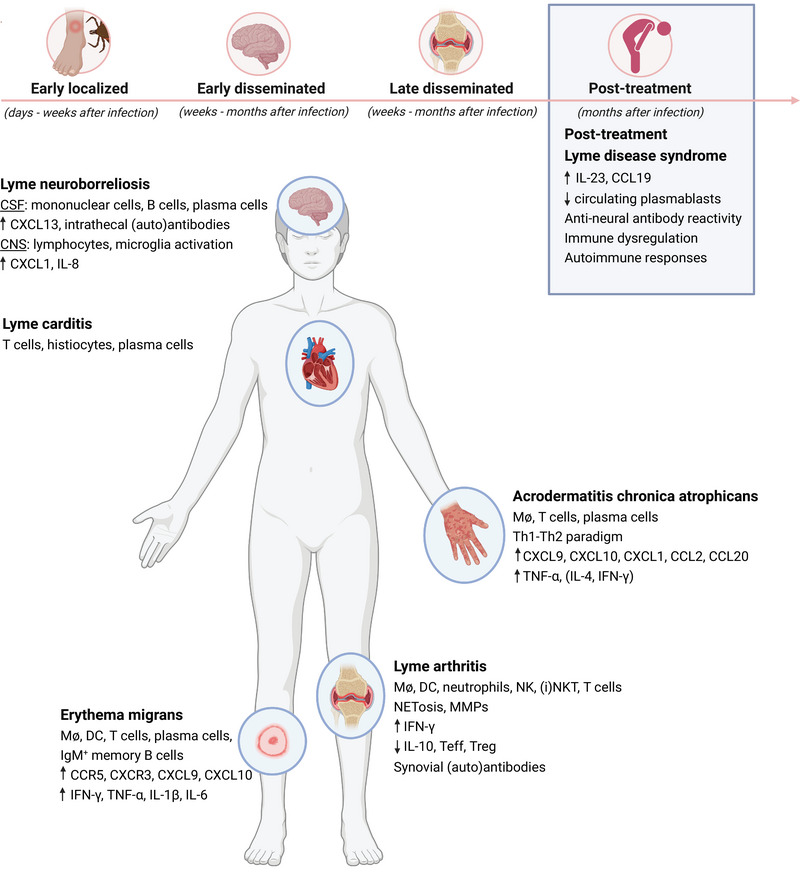
Clinical manifestations and inflammatory signatures of Lyme borreliosis. Clinical manifestations are divided into early localized disease (erythema migrans), early disseminated disease (Lyme carditis and Lyme neuroborreliosis), late disseminated disease (acrodermatitis chronica atrophicans and Lyme arthritis), and post‐treatment persisting complaints (post‐treatment Lyme disease syndrome).

Lyme borreliosis (LB), or Lyme disease, is the most common vector‐borne disease in the Northern Hemisphere caused by infection with extracellular spirochetes of the *Borrelia burgdorferi* sensu lato (Bbsl) genospecies transmitted by hard‐bodied *Ixodes* ticks [1]. The majority of LB cases are caused by three genospecies: *Borrelia burgdorferi* sensu stricto (*B. burgdorferi* ss) in the USA, *B. afzelii* and *B. garinii* in Europe and Asia [2]. Depending on the geographical region, Bbsl genospecies are transmitted to humans by different *Ixodes* tick species [3, 4]. Even though humans are accidental hosts, the incidence of LB has been increasing over time, partly due to climate change [5, 6]. The estimated LB incidence increased to 476,000 cases in the USA and 250,000 cases in Western Europe annually [7, 8]. LB has a broad clinical spectrum which can be arbitrarily divided into three stages: (1) early localized disease, characterized by an erythema migrans (EM) skin lesion; (2) early disseminated disease, characterized by two or more EM lesions and neurologic or cardiac involvement; (3) late or late disseminated disease, mainly characterized by arthritis or a chronic skin manifestation (acrodermatitis chronica atrophicans [ACA]) (Fig. 1) [1], [9]. Despite appropriate antibiotic treatment, approximately 4–10% of patients continue to experience symptoms such as fatigue, musculoskeletal pain, cognitive impairment, and other constitutional symptoms referred to as post‐treatment Lyme disease syndrome (PTLDS) [10, 11, 12]. The heterogeneous clinical presentation of LB has been linked to the wide range of Bbsl strains and the diversity in global epidemiology [4]. Seroprevalence of Bbsl‐specific antibodies increases during the course of infection, however, often fails to establish long‐term immunity [13]. Since Bbsl does not seem to excrete toxins that promote infection and disease, several clinical manifestations are indicated to be the result of an induced inflammatory response of the human host itself [14]. This review provides an overview of cellular and humoral immune responses elicited during infection with Bbsl and its potential to exacerbate clinical symptoms in LB patients.

## Clinical manifestations and inflammatory signatures

### Early localized disease

Early localized infection with Bbsl is characterized by EM lesions, a commonly expanding round or oval‐shaped skin rash at the tick bite site [1, 15]. This typical skin lesion is only detected in nonreservoir hosts due to a lack of local tolerance to Bbsl [4, 16, 17]. EM lesions are histologically characterized by perivascular and interstitial infiltrates of T cells, macrophages, dendritic cells, occasional plasma cells, and less commonly neutrophils and mast cells [18, 19, 20]. Recently, IgM memory B cells were detected in EM lesions, which are suggested to exhibit an antigen‐presenting cell function to T cells and mediate local antibody production that may be important for early protective humoral immunity [21, 22, 23]. Analysis of epidermal blister fluid has shown that more than 80% of the T cells in EM skin lesions express C‐C chemokine receptor type 5 and C‐X chemokine receptor 3 as well as IFN‐γ, suggesting a type 1 CD4^+^ T helper (Th1) cell response [24, 25, 26]. The presence of chemokine (C‐X‐C motif) ligand 9 (CXCL9) and CXCL10 subsequently contributes to the migration of these Th1 cells to the skin lesion [27]. Besides IFN‐γ, pro‐inflammatory cytokines TNF‐α, IL‐1β, and IL‐6 were also detected in EM lesions (Fig. 1) [2]. Comparing chemokine and cytokine mRNA levels in EM lesions from LB patients in Europe and the USA, infected with *B. afzelii* and *B. burgdorferi* ss, respectively, has revealed significantly higher mRNA levels for chemokines associated with macrophage activation and Th1 cell recruitment in patients from the USA [26]. Furthermore, it has been demonstrated that human monocyte‐derived macrophages stimulated with *B. burgdorferi* ss in vitro secrete higher levels of IL‐8, C‐C motif chemokine ligand 3 (CCL3), CCL4, IL‐6, IL‐10, and TNF compared to *B. garinii* or *B. afzelii* stimulation [28]. It is therefore suggested that the greater inflammatory potential of *B. burgdorferi* ss results in a more severe disease manifestation detected in USA patients compared with European patients infected with other Bbsl genospecies [28, 29, 30].

### Early disseminated disease

The early localized phase of LB can be followed by dissemination of spirochetes into deep tissues, including the heart, joints, and CNS [9]. Dissemination of Bbsl to the heart can cause Lyme carditis, a rare manifestation characterized by conduction abnormalities in the heart, mild myopericarditis, tachyarrhythmias, and myocardial dysfunction [31]. The incidence of Lyme carditis is more frequent in the USA (4–10%) compared with Europe (0.3–4%) and is mostly detected in patients between the ages of 20 and 40 years with an overall male predominance [32, 33, 34]. In Lyme carditis patients it was shown that predominantly the atrioventricular and sinoatrial nodes and the myocardium were infiltrated with T cells, histiocytes, and plasma cells (Fig. 1) [35], [36]. In murine models, it has been shown that CD4^+^ T cells were the primary mediators of myocarditis [37]. Cardiac injury has been assumed to be caused by an exaggerated immune response [38].

In 10–15% of LB patients, Bbsl disseminates to the CNS and can cause neurological manifestations referred to as Lyme neuroborreliosis (LNB) [39], [40]. In the USA, the most common neurological manifestations caused by *B. burgdorferi* ss are lymphocytic meningitis and cranial neuropathy, of which the latter results in facial palsy [1], [40]. In Europe, LNB is often caused by *B. garinii* and is most commonly characterized by painful meningoradiculitis, also known as Garin–Bujadoux–Bannwarth syndrome, which can be associated with lymphocytic meningitis followed by cranial neuropathy of the extremities and cerebrospinal fluid (CSF) pleocytosis [41]. Partial paralysis of the extremities occurs in 75% of LNB patients. Cranial neurites, mononeuritis multiplex, and plexus neuritis may also occur after infection but are less frequently detected [40]. These neurological symptoms are largely attributed to the resulting host–immune response in the CNS [42]. In vivo mouse models have shown Bbsl infiltrates particularly in vascular, perivascular, and extravascular regions of the dura mater of the meninges of the brain, followed by an increase in leukocytes [43], [44]. In nonhuman primate models, spirochetes were mainly localized in the leptomeninges [45]. Abnormalities in the CSF of LNB patients, such as mononuclear pleocytosis, persistent plasma cells, and intrathecal synthesis of Bbsl‐specific antibodies are usually detected within 3 to 6 weeks after infection [46]. Furthermore, elevated levels of CXCL13 and a subsequent infiltrate of B cells in the CSF are suggested as potential biomarkers for LNB [47]. Furthermore, microglial activation is suggested to play an important role in the early onset of LNB by recruitment of peripheral immune cells promoting inflammatory responses contributing to disease pathology. Not only post‐mortem brain tissue of LNB patients has shown a widespread microglial activation along with lymphocytic infiltrates in the CNS (Fig. 1) [48], but also in vitro stimulation of primary mice and rhesus microglia with *B. burgdorferi* ss has shown their activation by secretion of proinflammatory cytokines and chemokines such as IL‐6, TNF‐α, IL‐8, CCL3, and CCL4 [49], [50]. Similar cytokine and chemokine release has been described for human primary microglia, amongst which CXCL‐1 and IL‐8 were upregulated the most [51]. Inoculation of *B. burgdorferi* ss into the cisterna magna of rhesus macaques, an in vivo model for acute LNB, has shown elevated levels of IL‐6, IL‐8, CCL2, and CXCL13 in the CSF as early as 1 week post‐inoculation. CCL2 and CXCL13 were mainly found in microglia, indicating that microglial activation could play an important role in the recruitment of peripheral immune cells promoting inflammatory responses contributing to early onset of LNB [52]. This might be accompanied by the disruption of the blood‐brain barrier by Bbsl, as was demonstrated in a 3D blood–brain barrier organoid model [53], and potentially by dysregulation of the blood–CSF barrier by the choroid plexus, as was previously also seen in infection with *Neisseria meningitidis*, *Streptococcus suis*, and Echovirus [54, 55, 56, 57]. As these studies are being performed with culture‐grown Bbsl expressing different surface proteins compared with mammalian‐adapted spirochetes, it remains elusive if these proinflammatory responses occur similarly in human tissues.

### Late or late disseminated disease

In Europe, ACA is the most common late and chronic manifestation of LB [4]. It is primarily associated with *B. afzelii* infection and can occur months to years after a tick bite [58, 59, 60]. ACA starts with a slight red bluish distinguished by inflammation and edema which over time can develop into an atrophic skin lesion. ACA is most commonly detected in adults between 40 and 50 years of age with a higher prevalence in women [61, 62]. Polyneuropathy is reported in 40–60% of ACA patients, caused by axonal degradation of the nerves located outside the brain and spinal cord, which primarily progresses at the distal parts of the extremities resulting in local sensory loss [60]. Additionally, patients can also experience rheumatologic symptoms [63]. The skin lesions of ACA are characterized by an infiltrate of T cells and plasma cells which can extend to the subcutis, high levels of CXCL9 and CXCL10, and intermediate levels of CXCL1, CCL2, and CCL20 (Fig. 1) [27, 64]. Contradictory results have been reported about the presence of IFN‐γ and IL‐4 in ACA. On the one hand, ACA lesions showed a restricted cytokine response consisting of mainly TNF‐α and IL‐4. The absence of IFN‐γ in ACA skin lesions could result in spirochetal persistence due to the lack of an extensive inflammatory response [19]. On the other hand, increased levels of IFN‐γ were described for ACA, but decreased IL‐4 levels compared with EM lesions, suggesting a more crucial role for IL‐4 in chronic ACA manifestations [27, 65].

Lyme arthritis (LA) is the primary late disseminated disease manifestation in the USA caused by *B. burgdorferi* ss, whereas in Europe LA is less frequently detected suggesting that *B. afzelii* and *B. garinii* are less arthritogenic [1]. Weeks to months after Bbsl joint infection, LA is initiated by enhanced cytokine and chemokine release by macrophages and dendritic cells, formation of neutrophil extracellular traps, the release of IFN‐γ by natural killer T cells, and secretion of matrix metalloproteinases by tissue resident cells such as chondrocytes (Fig. 1) [66]. A recent study has discovered a potential inflammatory role for *B. burgdorferi* ss peptidoglycan (PG^Bb^), an essential cell wall component, as exposure to PG^Bb^ induced inflammatory infiltrates in the joints of mice [67]. In most cases antibiotic treatment resolves LA symptoms, however, in a small number of patients synovitis worsens resulting in synovial hyperplasia referred to as post‐antibiotic arthritis [68]. Factors described to be associated with the development of post‐antibiotic arthritis are for example the presence of HLA‐DR isotype alleles that bind a specific epitope of Bbsl Outer surface protein A (OspA_165_–_173_), TLR‐1‐1805GG polymorphism, and infection with a more invasive ribosomal RNA intergenic spacer type 1 strain of *B. burgdorferi* ss [69, 70, 71]. These factors result in high amounts of IFN‐γ, low amounts of IL‐10, decreased ratios of regulatory T cells and effector T cells, and the development of autoantibodies (Fig. 1). Dysregulation of type I IFN responses have also been associated with the development of rheumatic diseases [72, 73]. Disease‐susceptible C3H/HeJ or C3H/HeN mice with a dominant Th1 response upon *B. burgdorferi* ss infection displayed reduced arthritis severity after anti‐IFN‐γ monoclonal antibody (mAb) therapy [74]. Targeting IL‐12, which plays a critical role in the induction of IFN‐γ responses, showed similar results in these mice [75]. Conversely, blocking Th2 responses with anti‐IL‐4 mAb in nonsusceptible BALB/c mice significantly increased joint swelling, indicating that arthritis severity can be attenuated by Th2 responses [74]. Nonetheless, this Th1–Th2 paradigm in arthritis susceptibility and resistance in mice has been challenged as C3H/HeJ IFN‐γ and DBA/2J IL‐4 knockout mice developed arthritis of comparable severity to their wild‐type counterparts [76]. Similar results were found in IFN‐γ receptor‐deficient nonsusceptible 129/SvEv mice as well as C57BL/6 and BALB/c mice deficient for either IL‐4 or IL‐4 receptor alpha chain [77, 78]. While antibiotic therapy effectively resolves arthritis in wild‐type mice infected with Bbsl, IL‐10 knockout mice resemble human post‐infectious LA with low spirochetal burden, T‐cell infiltration of the synovium, and elevated IFN‐γ serum levels. In this model, T cells maintain their activated state despite undetectable pathogens loads in joint tissue [79]. RNA sequencing of synovial tissue of post‐infectious LA patients demonstrated excessive expression of IFN‐γ related genes which inversely correlated with expression of genes related to the repair of damaged tissue [80]. Multiple lymphocyte subsets, that is, CD4^+^ T cells, cytotoxic CD8^+^ T cells, double‐negative T cells, and NK cells, were shown to contribute to this IFN‐γ profile [81]. Although IFN‐γ production by (invariant) natural killer T cells is shown to have a protective function in controlling spirochete invasion of joint and heart tissue [82, 83], excessive IFN‐γ responses result in unfavorable disease outcomes [84]. Patients with post‐infectious LA typically exhibit positive responses to immunosuppressive drug treatments, suggesting that this particular form of inflammatory arthritis is no longer caused by active infection. Altogether this indicates that an excessive and/or maladaptive host inflammation is a critical factor for post‐antibiotic arthritis pathology [85].

### Post‐treatment Lyme disease syndrome

Although the majority of LB patients fully recover after antibiotic treatment, long‐lasting symptoms after treatment have been reported in approximately 4–10% of LB patients [11]. Long‐term post‐infection symptoms such as fatigue, musculoskeletal pain, and cognitive impairment have not only been described for LB but also for other infectious diseases [86], [87]. As spirochetal numbers are reduced to undetectable limits within weeks to months post‐infection and retreatment with antibiotics does not show any benefit in symptom relief [1, 88, 89], it is hypothesized that long‐lasting symptoms in PTLDS patients might not be attributed to persisting infection but to dysregulated inflammatory responses. In particular elevated serum levels of IL‐23 and lower levels of circulating plasmablasts during the acute phase as well as persisting serum levels of CCL19 have been linked to the development of PTLDS (Fig. 1) [90, 91, 92]. Furthermore, IL‐23 and CCL19 have been shown to drive pathogenic Th17 responses in an encephalomyelitis mouse model [93]. However, since IL‐17 [94], [95] and IL17 receptor [96] deficient mice develop Lyme arthritis and carditis after Bbsl infection similarly compared with their wild‐type counterparts, the detrimental role of IL‐17 in PTLDS remains controversial.

## Disruption of long‐term immunity

**Figure 2:**
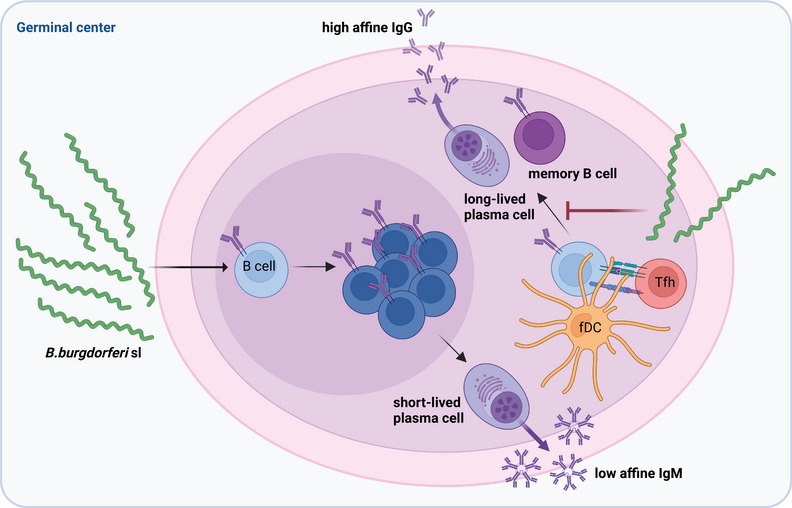
Inhibition of long‐term immunity by *Borrelia burgdorferi* sensu stricto. Accumulation of *B. burgdorferi* ss in the lymphoid structures in mice induces B cell proliferation and differentiation to short‐lived antibody‐secreting plasma cells producing low affine IgM but inhibits the formation of long‐lived memory B cells and plasma cell formation induced by follicular dendritic cells and follicular helper T cells. This results in involution of the germinal center, decreased high affine IgG production, and lack of immune memory.

The generation of high‐affinity antibodies and the formation of memory B cells are essential to establish long‐term immunity against pathogens [97]. Even though Bbsl‐specific IgM and IgG seroprevalence has been detected up to multiple years after treatment, antibody persistence was not associated with persisting infection and was independent of clinical resolution [98, 99]. Ongoing inflammation, as a result of a complex interplay between host and immune responses and Bbsl immune evasion strategies (extensively reviewed elsewhere) [100], is therefore suggested to be associated with clinical symptoms following Bbsl infection rather than the pathogen itself. As B cells and CD4^+^ T cells are crucial for the induction of high‐affinity class‐switched antibody responses, their dysfunction is implied in the absence of long‐lasting immunity in LB patients. Bbsl infected C57BL/6J (B6)‐Rag1 knockout mice, which lack T‐ and B cells, developed severe arthritis and myocarditis after reconstitution with either pan T cells or only CD4^+^ T cells, indicating a minor role for T cells in reducing the duration of active infection and the severity of LB [101]. Mice lacking both conventional‐, and nonconventional T cells (B6‐TcrβTcrδ knockout mice), but possessing B cells, were able to resolve mild carditis and synovitis, affirming the importance of B cells in disease resolution [37]. It was shown that *B. burgdorferi* ss accumulated in the lymphoid structures, inducing T‐cell independent B‐cell proliferation and differentiation to antibody‐secreting plasma cells (Fig. 2) [102], [103]. Even though B cells were robustly activated, a time‐course study demonstrated that germinal centers (GC), essential for affinity maturation and immune memory generation, were short‐lived and failed to produce long‐lived plasma or memory B cells [13], [104]. Despite the lack of long‐lived plasma cells, low affine IgM was still produced throughout infection which only provided passive immune protection and did not prevent Bbsl dissemination into solid tissues [104, 105]. Follicular helper T cells, necessary for GC reactions, were effectively primed in *B. burgdorferi* ss infected mice resulting in high‐affinity IgG production. This T‐cell‐dependent IgG production was reversed later in disease coinciding with the involution of GC [106]. Possibly, the presence of *B. burgdorferi* ss in the lymph nodes blocks the formation of long‐lived GC and thus long‐lived memory formation (Fig. 2). In addition to the lack of immune memory after *B. burgdorferi ss* infection, mice were also incapable of robustly inducing antibodies in response to influenza immunization, indicating a temporal immunosuppression caused *by B. burgdorferi* ss [106]. The molecular targets of *B. burgdorferi* ss and factors driving the involution of GCs remain to be identified. Since mice are natural Bbsl reservoirs and humans are not, these findings should be carefully interpreted. Compared to mice, the human B‐cell response following acute Bbsl infection remains understudied. A strong induction of mainly plasmablasts and immature antibody‐secreting cells was detected in the circulation of a subpopulation of LB patients before antibiotic treatment [92]. Although not significant, this plasmablast induction was also found in a subset of patients from another study in Europe, investigating the B‐cell populations of LB patients after the start of antibiotic treatment [107]. Similar to mice, B cells seemed to offer at least partial protection against severe LB in humans, as high plasmablast levels correlated with Bbsl resolution [92], [108]. Nonetheless, despite strong plasmablast induction in some LB patients, abnormalities in the B‐cell memory responses were found. Instead of an increase in the usually dominating CD27^+^ IgD‐ memory B cells, an increase in autoimmunity‐associated CD27^−^IgD‐ memory B cells was found 1 month post‐infection [92, 109, 110]. Furthermore, transcriptomics of peripheral blood mononuclear cells of LB patients in the USA revealed an absence of developmental B‐cell pathways [111]. In vitro stimulation of human peripheral blood mononuclear cells with Bbsl resulted in inhibition of antigen presentation by decreased expression of CD74, HLA‐DM, and HLA‐DR which could possibly be an explanation for the lack of functional adaptive immune responses against Bbsl [112]. Furthermore, rs1061632 major allele (T) variant linked to upregulation of the mammalian target of rapamycin pathway [113] and cytokine quantitative trait locus *TLR1‐6‐10* [114] were recently identified to be associated with LB susceptibility suggesting that immunogenetic variation may play an important role in LB pathogenesis. Moreover, the LB transcriptome showed a relatively high overlap with three different immune‐mediated chronic diseases indicating an inflammatory response to *B. burgdorferi* ss, rather than resolution [111]. Interestingly, corroborating the increase in autoimmunity‐associated CD27^−^IgD‐ memory B cells, the LB transcriptome showed a relatively high overlap with three different immune‐mediated chronic diseases indicating an inflammatory response to *B. burgdorferi* ss, rather than resolution [111].

## Development of autoimmunity

**Figure 3:**
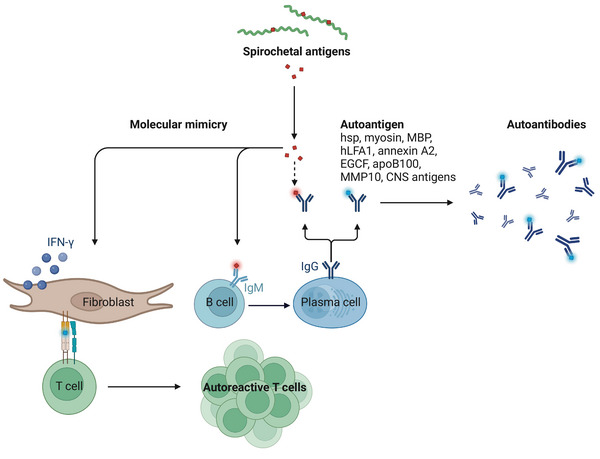
Development of autoantibodies and autoreactive T cells via molecular mimicry. High similarity between spirochetal antigens and autoantigens heat shock protein (hsp) family, myosin, myelin basic protein (MBP), human leukocyte function‐associated antigen‐1 (hLFA1), annexin A2, endothelial growth cell factor (EGCF), apolipoprotein B‐100 (apoB100), matrix metalloproteinase‐10 (MMP10), and CNS antigens result in the production of autoantibodies due to molecular mimicry. In the presence of *Borrelia burgdorferi* ss and IFN‐γ fibroblasts and fibroblast‐like cells present autoantigens to naïve CD4^+^ T cells resulting in the proliferation of autoreactive T cells. Autoreactive immune responses potentially contribute to Lyme borreliosis disease manifestations, such as Lyme arthritis, Lyme neuroborreliosis, and post‐treatment Lyme disease syndrome.

LB has been associated with autoimmunity as clinical manifestations resemble those of autoimmune disorders. Mechanisms such as molecular mimicry, epitope spreading, bystander activation, original antigenic sin, polyclonal B‐ and T cell activation, and apoptosis of antigen‐presenting cells are associated with the development of autoimmunity [115], [116]. In LB, molecular mimicry is the most well‐studied mechanism due to the high similarity of Bbsl antigens and self‐antigens which can result in the induction of autoreactive lymphocytes and antibodies (Fig. 3) [117]. In LA, autoreactivity against the heat shock protein (hsp) family, myosin, myelin basic protein (MBP), human leukocyte function‐associated antigen‐1 (hLFA‐1), annexin A2, endothelial growth cell factor (EGCF), apolipoprotein B‐100 (apoB100), and matrix metalloproteinases‐10 (MMP10) have been correlated to disease pathology [118, 119, 120, 121, 122, 123]. Fibroblasts harboring Bbsl or primed with PG^Bb^ and IFN‐γ in vitro presented autoantigens LFA‐1 and extracellular matrix peptides in major histocompatibility complex II inducting autoreactive CD4^+^ T cell activation which may perpetuate inflammation and disease pathogenesis [119, 124]. In contrast to anti‐Bbsl antibodies of the IgG1 and IgG3 isotype, autoantibodies found in LA patients were mainly of the IgG4 isotype and correlated with synovial pathology [125]. The implication of IgG4 autoantibodies in LA pathology has furthermore been supported by a recent study showing that loss of function of the inhibitory Fcγ‐receptor IIb, binding IgG4 complexes, resulted in lower Bbsl pathogen burden in the directly infected joint and distal joint in humanized mice [126]. Similarly, as seen in LA, autoreactive immune responses against antigens in the brain, spinal cord, dorsal root ganglia, gangliosides, and peripheral nerve axons have been detected in LNB and PTLDS patients, potentially contributing to neurological abnormalities seen in these patients [127, 128]. Additionally, case reports have described the relationship between LB and the development of autoimmune diseases such as dermatomyositis, Guillain‐Barre syndrome, Sjögren's syndrome, Still's disease, and systemic lupus erythematosus. LB could have provided a secondary signal, also known as the adjuvant effect, required for the initiation of these autoimmune disorders. The development of autoantibodies during infection is, however, not specific to LB as this phenomenon is also detected in other infectious diseases such as COVID‐19, HIV, tuberculosis, and malaria [129, 130]. Although autoantibody‐mediated pathology in autoimmune diseases is well described, their pathogenic role during‐ and post‐infection remains elusive.

## Conclusion

LB presents a broad clinical spectrum with varying manifestations driven by the host–immune response. Inflammatory responses triggered by Bbsl infection play a central role in the pathogenesis of LB, with diverse cytokines, chemokines, and immune cell subsets orchestrating the disease process. Failure to establish long‐term immunity against Bbsl, as well as emerging evidence for a potential link between LB and the development of auto‐inflammatory responses or autoimmunity, highlights the complexity of host‐pathogen interactions. Future research should focus on elucidating the mechanisms underlying host–immune responses and their role in LB disease progression. A comprehensive understanding of the immunopathogenesis of LB could be crucial for the development of targeted interventions and the mitigation of its clinical burden on affected individuals.

## Conflict of interest

The authors declare no commercial or financial conflict of interest.

## Author contributions

Marijn E. Snik, Noor E.I.M. Stouthamer, and Melissa M.J. van Gool conceptualized the review framework. Marijn E. Snik and Noor E.I.M. Stouthamer drafted the manuscript. Melissa M.J. van Gool and Joppe W. Hovius edited all versions and Melissa M.J. van Gool drafted the figures.

### Peer review

The peer review history for this article is available at https://publons.com/publon/10.1002/eji.202451063

**Abbreviations**
ACA: acrodermatitis chronica atrophicans
Bbsl: *Borrelia burgdorferi* sensu lato
CSF: cerebrospinal fluid
CXCL9: C‐X‐C motif ligand 9
EM: erythema migrans
GC: germinal centers
LA: Lyme arthritis
LB: Lyme borreliosis
LNB: Lyme neuroborreliosis
mAb: monoclonal antibody
PTLDS: posttreatment Lyme disease syndrome

## Data Availability

Data sharing is not applicable to this article as no new data were created or analyzed in this study.
